# *From Lab to Lake* – Evaluation of Current Molecular Methods for the Detection of Infectious Enteric Viruses in Complex Water Matrices in an Urban Area

**DOI:** 10.1371/journal.pone.0167105

**Published:** 2016-11-23

**Authors:** Mats Leifels, Ibrahim Ahmed Hamza, Marion Krieger, Michael Wilhelm, Martin Mackowiak, Lars Jurzik

**Affiliations:** 1 Ruhr-University Bochum, Department of Hygiene, Social- and Environmental Medicine, Bochum, Germany; 2 Environmental Virology Laboratory, Department of Water Pollution Research, National Research Centre, Cairo, Egypt; 3 University of Duisburg-Essen, Faculty of Chemistry, Biofilm Centre – Aquatic Microbiology, Essen, Germany; Sidra Medical and Research Center, QATAR

## Abstract

Quantitative PCR methods are commonly used to monitor enteric viruses in the aquatic environment because of their high sensitivity, short reaction times and relatively low operational cost. However, conclusions for public health drawn from results of such molecular techniques are limited due to their inability to determine viral infectivity. Ethidium monoazide (EMA) and propidium monoazide (PMA) are capable to penetrate the damaged or compromised capsid of the inactivated viruses and bind to the viral nucleic acids. We assessed whether dye treatment is a suitable approach to improve the ability of qPCR to distinguish between infectious and non-infectious human adenovirus, enterovirus and rotavirus A in surface water of an urban river and sewage before and after UV disinfection. Like the gold standard of cell culture assays, pretreatment EMA-/PMA-qPCR succeeded in removing false positive results which would lead to an overestimation of the viral load if only qPCR of the environmental samples was considered. A dye pretreatment could therefore provide a rapid and relatively inexpensive tool to improve the efficacy of molecular quantification methods in regards to viral infectivity.

## Introduction

Outbreaks of waterborne enteric viruses are a major public health concern. The presence of even a few infectious viral particles in large volumes of environmental water which are used for drinking water production or for recreational purposes can pose a threat to the consumer and therefore public health [1]. So far, almost 150 different types of viruses are known which cause a variety of illnesses and diseases in human and can be found in the aquatic environment due to sewage contamination [2].

Analytical methods for virus detection in environmental samples continue to rely on long established methods like animal tissue culture, quantitative polymerase chain reaction (qPCR) and the integrated cell culture PCR. Even though cell culture remains the gold standard for the detection of viral infectivity, the cell lines used are not specific for certain virus (e.g. norovirus) which makes it necessary to combine it with a follow-up molecular or immunological assay for confirmation [3]. Since it is time consuming, labor-intensive and expensive, cell culture cannot be used as a routine and robust detection tool.

The qPCR is highly specific, relatively cost effective as well as adaptable and provides fast results. However, it lacks the ability to determine viral infectivity. Inhibitors which might be co-concentrated during processing of environmental samples are also known to interfere with the polymerase and therefore may limit the use of qPCR for virus analysis [4]. The integrated cell culture qPCR (ICC-qPCR) is capable to distinguish between viable and non-viable viruses. Its application has been described for a broad spectrum of aquatic human pathogenic viruses like enterovirus, hepatitis E virus [5], adenovirus and rotavirus [6–9]; however, it is still time consuming, labor-intensive and expensive. Moreover, the lack of cell lines for the detection of human-pathogenic norovirus limits the use of ICC-qPCR. Recently, few trials to propagate norovirus in 3D cell culture settings have been succeeded which may help in this context [10].

The treatment of samples inactivated by heat, chlorine and UV light as well as with enzymes like RNase and DNase show efficient exclusion of false positive signals in follow-up qPCR, but if the viral capsid was still intact, no correlation between viral infectivity and qPCR results could be found [11, 12].

A promising approach to determine viral infectivity is the viability PCR (vPCR), the application of the ethidium monoazide (EMA) and propidium monoazide (PMA) prior to qPCR or reverse transcription qPCR. Both reagents contain a photo-inducible azide group that covalently binds to nucleic acids after exposure to light with a specific wavelength which results in a significantly decreased signal in a subsequent qPCR due to the inhibition of the polymerase [13]. The usage of PMA and EMA has been proposed for the selective detection of a broad spectrum of organisms including bacteria [14–18], fungi [19, 20], various protozoa including incorporated bacteria [21–23] and nematode eggs [24]. The application of the method for the distinction between infectious and non-infectious viruses has been investigated thoroughly in lab scale [25–29]. Its application also has been proposed in food safety [30, 31] and for the detection of enteric viruses in the environment [32–35].

The presented work aims to assess the suitability of vPCR to selectively detect infectious and non-infectious human adenovirus (HAdV), enterovirus (EV) and rotavirus (RV) in complex water matrices like surface water from an urban river in the metropolitan Rhine-Ruhr Region, North-Rhine Westphalia, Germany and partly treated sewage water prior and after ultraviolet light treatment just before its release into the environment.

The correlation of cost—both monetary and time—and benefit of the compared methods is also evaluated in order to assess which technique is to be recommended for the robust and cost effective determination of viral infectivity in complex water matrices.

## Material and Methods

### Collection and Concentration of Environmental Water Samples for the Viral Analysis

**Fig 1:**
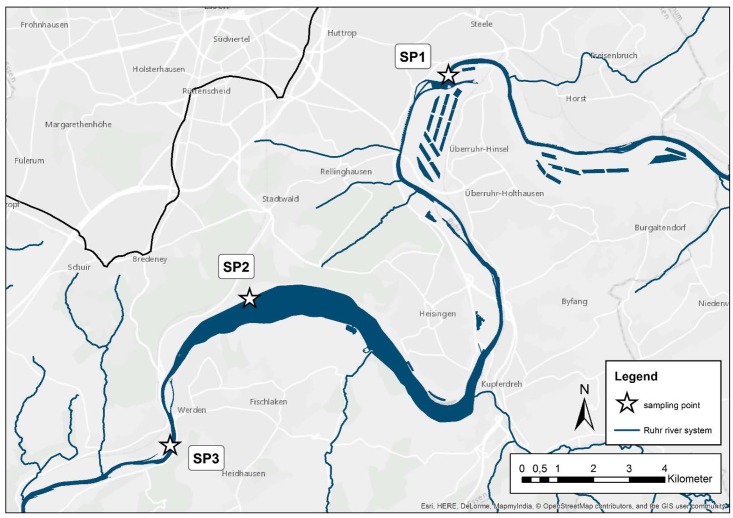
Sampling sites for the surface water extraction at Ruhr River, North Rhine Westphalia, upstream, downstream and at the putative recreational Lake Baldeney in the city of Essen, Germany.

Prior to the virus analysis, 53 surface water samples (10 liter) have been collected between May and September 2015 from different sampling sites (depicted in Fig 1; SP1–51°26'30.4"N 7°04'09.4"E, SP2–51°24'22.0"N 7°01'11.2"E, SP3- 51°22'51.5"N 6°59'51.1"E) at the urban Ruhr River in North Rhine Westphalia, Germany and analyzed for their physico-chemical parameters as described before [36].

Sample collection was approved by the German Federal Ministry of Education and Research (BMBF project number 02WRS1283A to J) and we confirm that the field studies did not involve endangered or protected species.

In parallel, 26 samples have been taken between September and October 2015 from wastewater treatment facilities before and after UV treatment as a final disinfection step. After transfer to the laboratory, the samples have been concentrated following the VIRADEL method as previously described [37].

Following the VIRADEL protocol, all water samples (pH 6.69–8.15, temperature 12.2–19.7°C) have been spiked with murine norovirus (approximately 3.00E+02 genome equivalent copies) as an external process control in order to evaluate the recovery rate of the enrichment (50%– 75%), the extraction and amplification of viral nucleic acids (data not shown). In brief MgCl_2_ was added to the sample with a final concentration of 0.05 M to stabilize the viral capsids. The pH was adjusted at 3.5 with 1 N HCl and a negatively charged nitrocellulose membrane with 0.45 μm pore size and a diameter of 142 mm was used for the filtration and viral adsorption. Elution and recovery of the bound viruses was performed by eluting the filter with a non-organic elution buffer (0.005 M KH_2_PO_4_, 0.5 M NaCl, 0.1% (v/v) Triton x-100, pH 9.2) and a reconcentration with 12.5% polyethylene glycol 6000 (PEG) and 2.5% NaCl at 4°C overnight. Concentrated samples were stored at -20°C until analysis by molecular or culture based methods.

### Dye Treatment of the Concentrated Environmental Samples

The treatment of the samples has been performed according to our previous study [25] with adjustments based on the finding of Prevost, Goulet [32]. Stock solutions of EMA and PMA (both Biotium Inc, Hayward, CA) have been reconstituted with 20% dimethyl sulfoxide (DMSO; Sigma-Aldrich Co., St. Louis, MO) up to a concentration of 10 mM and stored at -20°C. Aliquots of both dyes with a working concentration of 1 mM were freshly prepared. PMA and EMA were added in a final concentration of 0.04 mM per reaction to 200 μl of environmental samples diluted with phosphate buffered saline (Sigma-Aldrich Co., St. Louis, MO), mixed gently and incubated in the dark and on ice for 30 min. Preliminary experiments showed that a 1:5 dilution helped to reduce interference of the assay by the presence of particles. Photo-activation was performed by transferring the samples to the PhaST Blue System (IUL, Barcelona, Spain) and exposure for 15 min with 100% intensity and subsequent extraction of total viral genome.

### Extraction of Viral Nucleic Acids

Viral DNA and RNA have been co-extracted from 200 μl of the virus suspension using the QIAmp DNA Blood Mini Kit (Qiagen, Hilden, Germany) according to the manufacturer’s instructions. Total nucleic acids were eluted in 100 μl elution buffer and stored at -20°C.

### Quantitative Real Time PCR (qPCR)

Standards containing DNA and RNA have been produced according to our previous study [37]. All primers used in this study for the detection of enteric viruses in the environment are listed in Table 1.

**Table 1:** Sequences, size of the amplicon and target region of the primer and probe sets used for the qPCR detection of human Adenovirus (HAdV), enterovirus (EV), murine norovirus (MNV) and rotavirus (RV) in environmental samples.

Virus	Primer	Sequence 5’-3’	Size (bp)	Target	Reference
**HAdV**	AQ 1	GCC ACG GTG GGG TTT CTA AAC TT	132	Hexon	[38]
	AQ 2	GCC CCA GTG GTC TTA CAT GCA CAT C			
	HAdV P	[6FAM] TGC ACC AGA CCC GGG CTC AGG TAC TCC GA [BHQ1]			
**EV**	EV 444	CCT CCG GCC CCT GAA TG	178	5’-UTR	[37]
	EV 621	ACC GGA TGG CCA ATC CAA			
	EV P	[6FAM] ACG GAC ACC CAA AGT CGG TTC CG [BHQ1]			
**RV**	F	ATG GAT GTC CTG TAC TCC TTG TCA AAA	128	VPN	[39]
	R	TTC CTC CAG TTT GRA AST CAT TTC C			
	Rota P 1	[6FAM] ATA ATG TGC CTT CGA CAA T-[MGBNFQ]			
	Rota P 2	[6FAM] AAT ATA ATG TAC CTT CAA CAA T-[MGBNFQ]			
**MNV**	TMP 1	AGA GGA ATC TAT GCG CCT GG	92	ORF2	[40]
	TMP 2	GAA GGC GGC CAG AGA CCA C			
	TMP	[6FAM] GCC ACT CCG CAC AAA CAG CCC [BHQ1]			

For RNA viruses, the High Capacity cDNA Reverse Transcription Kit (Thermo Fischer, Waltham, MS) was used to produce cDNA, following manufacturers’ protocol. The qPCR was performed using the Takyon no ROX qPCR Mastermix (Eurogentec, Liège, Belgium) in a 20 μl reaction volume containing 5 μl (2 μl of cDNA) of nucleic acid template, 0.25 μM of both forward and reverse primers and 1 μM of specific probe. The cycling conditions for all viruses have been described by Hamza, Jurzik [37]. The Rotorgene 6,000 qRT cycler system was utilized for amplification and detection. The limit of detection (LD) in copies per qPCR reaction was determined via serial dilution and calculated to be 5 genomic copies per liter for HAdV, MNV and RV and 50 for EV.

### qPCR Inhibition Control

In addition to the external process control murine norovirus (MNV; which has been recovered after treatment in rates between 50–75% in all samples (data not shown)), an amplification control has been performed for randomly selected positive river water samples as well as all samples negative for one or more viruses. For this control, the samples have been spiked with extracted nucleic acids of approximately 3.00E+02 genome copies of the respective DNA standard [37] prior to molecular quantification and the results have been compared to the original concentration.

### Infectivity Assay of qPCR Positive Enteric Viruses

For the detection of human adenovirus, the human lung adenocarcinoma cell line A549 was utilized [25], enterovirus and rotavirus were both propagated on African rhesus monkey kidney cells MA-104, which have been described as being suitable to determine viral infectivity of both environmental EV and RV [41]. The cells were propagated in Dulbecco’s MEM (DMEM; Sigma-Aldrich Co., St. Louis, MO) supplemented with 10% heat inactivated fetal bovine serum (FBS) and 1% penicillin-streptomycin for A549 and additional 1% non-essential amino acids and 1% l-glutamine for MA-104.

Cells for the detection of enteric viruses were transferred to 48-well tissue culture plates at a density of around 5.0E+04 cells per well. After 48 hours at 37°C and in the presence of 5% CO_2_, the cell culture medium containing FBS was removed, the wells were washed with PBS and then infected with 50 μl of the sample in serial dilution previously prepared in culture medium without FBS. Samples were inoculated for 90 min before being aspirated and 300 μl of maintenance medium with 2.5% FBS was added and incubated for 5 days for HAdV, RV and EV. During this incubation time, the cells were monitored for the occurrence of cytopathic effect (CPE) and the viral concentration calculated following the method of Reed and Muench [42] and expressed as TCID_50_/ml.

### Integrated Cell Culture qPCR

Following the protocol from a previous study [43], cells for the evaluated enteric viruses have been propagated as described above, transferred to 12-well plates and incubated for 2 days or until a confluent cell monolayer was obtained. The supernatant was removed and the cells were washed with PBS. Subsequently, the cells were inoculated in duplicates on two separate plates for 90 min at 37°C and 5% CO_2_ with reconcentrated environmental water samples. Cells were then incubated at the same conditions for 5 days and checked for CPE daily. After three cycles of freezing and thawing, nucleic acids from the cell lysate were extracted and viral genome copies were determined by qPCR. A 10-fold or more increase of the nucleic acid copy number was considered to indicate the presence of infectious enteric viruses.

### Statistical Analysis

Concentrations of viral genomic copies are described using genomic copies per liter (GC/l) for (RT-) qPCR, EMA-qPCR and PMA-qPCR and tissue culture infectivity dose for 50% of the exposed cells (TCID_50_) for the cell culture based assays [42]. Concentrations per liter are calculated based on qPCR results considering treatment recovery rates previously described [37]. Samples containing values lower than 2-fold the limit of detection are referred to as not detectable (nd). All statistical analysis and visualizations are conducted using Microsoft excel 2013 (Microsoft Inc., Redmond, WS, USA). Unprocessed values for each virus can be found in Tables A-C in S1 File.

## Results

### Molecular Quantification and Detection of Humane Adenovirus (HAdV)

**Fig 2:**
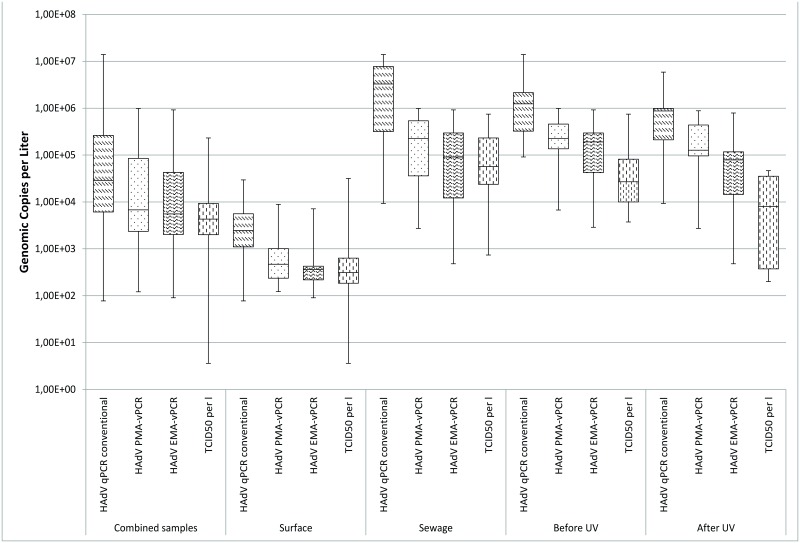
Boxplot comparison of the calculated concentration for human. Adenovirus originating from surface waters and sewage waters before and after UV treatment as well as total samples.

As shown in Table 2, pretreatment with the intercalating reagents lead to considerably lower rates for both EMA (29/79) and PMA (41/79). Before treatment, HAdV could be detected in a large part of the surface water samples (45/53) with median concentration of 2.45E+03 GC/l and in almost 90% of the combined sewage water samples (25/26) with a median concentration of 3.29E+06 GC/l as depicted in Fig 2.

**Table 2:** Occurrence of HAdV, EV and RoV in the combined, surface, total sewage water samples and before and after UV treatment.

		**qPCR**		**PMA vPCR**		**EMA vPCR**		**Cell Culture**	
**HAdV**	all samples (n = 79)	70	89%	41	52%	29	37%	41	52%
	surface water (n = 53)	45	84%	21	40%	11	21%	28	53%
	sewage water (n = 26)	25	96%	20	77%	18	69%	13	50%
	before UV (n = 13)	13	100%	12	92%	11	85%	9	69%
	After UV(n = 13)	12	93%	8	62%	7	54%	4	31%
**EV**		**RT-qPCR**		**PMA RT-vPCR**		**EMA RT-vPCR**		**Cell Culture**	
	all samples (n = 79)	13	17%	6	8%	6	8%	10	13%
	surface water (n = 53)	6	11%	2	4%	2	4%	5	9%
	sewage water (n = 26)	7	27%	4	15%	4	15%	5	15%
	before UV(n = 13)	6	46%	4	31%	4	31%	5	39%
	after UV(n = 13)	1	8%	Nd	-	nd	-	nd	-
**RV**		**qPCR**		**PMA RT-vPCR**		**EMA RT-vPCR**		**Cell Culture**	
	all samples (n = 79)	15	19%	3	4%	nd	-	8	10%
	surface water (n = 53)	10	19%	2	4%	nd	-	6	11%
	sewage water (n = 26)	5	19%	1	4%	nd	-	2	8%
	before UV (n = 13)	10	77%	1	8%	nd	-	6	46%
	after UV (n = 13)	nd	-	nd	-	nd	-	nd	-

EMA and PMA pretreatment resulted in lower detection rates and lower median GC/l in both surface (EMA 11/53; PMA 21/53) and sewage water samples (EMA 18/26; PMA 20/26).

UV disinfection was not efficient for HAdV inactivation based on qPCR results since all samples were positive before (13/13) and the majority after treatment (12/13). However, as seen in Table 3, utilization of both EMA and PMA resulted in lower occurrence in both UV inflow (EMA 11/13; PMA 12/13) and outflow (EMA 7/13; PMA 8/13).

**Table 3:** Calculated concentration of viral nucleic acids (GC/l) or infectious viruses (TCID_50_/l) before and after UV-treatment of the sewage water samples.

	Molecular methods				Cell culture assay
		**qPCR (GC/l)**	**vPCR PMA (GC/l)**	**vPCR EMA (GC/l)**	**Cell culture TCID**_**50**_
**HAdV**	**median before UV**	1.24E+06	2.27E+05	1.91E+05	2.69E+04
	**min**	9.11E+04	6.72E+03	2.88E+03	3.74E+03
	**max**	1.40E+07	9.85E+05	9.20E+05	7.46E+05
	**median after UV**	8.72E+05	1.27E+05	7.88E+04	8.03E+03
	**min**	9.29E+03	2.72E+03	4.79E+02	2.00E+02
	**max**	5.88E+06	8.80E+05	7.89E+05	4.64E+04
**EV**		RT- **qPCR (GeC per l)**	**vPCR PMA (GeC per l)**	**vPCR EMA (GeC per l)**	**Cell culture TCID**_**50**_ **PFU per l(confirmed by ICC-PCR/ICC-RT-PCR**
	**median before UV**	8.63E+03	5.81E+03	4.46E+03	2.02E+04
	**min**	3.50E+03	4.33E+02	1.90E+03	1.36E+04
	**max**	1.83E+04	6.48E+03	1.72E+04	2.94E+04
	**median after UV**	6,00E+03*	nd	nd	nd
	**min**		nd	nd	nd
	**max**		nd	nd	nd
**RV**		RT- **qPCR (GeC per l)**	**vPCR PMA (GeC per l)**	**vPCR EMA (GeC per l)**	**Cell culture TCID**_**50**_ **PFU per l (confirmed by ICC-PCR/ICC-RT-PCR**
	**median before UV**	1.30E+04	2.18E+04*	nd	6.11E+04
	**min**	5.01E+02		nd	2.94E+04
	**max**	4.75E+04		nd	9.28E+04
	**median after UV**	nd	nd	nd	nd
	**min**	nd	nd	nd	nd
	**max**	nd	nd	nd	nd

* = only one sample positive

### Molecular Quantification and Detection of EV

**Fig 3:**
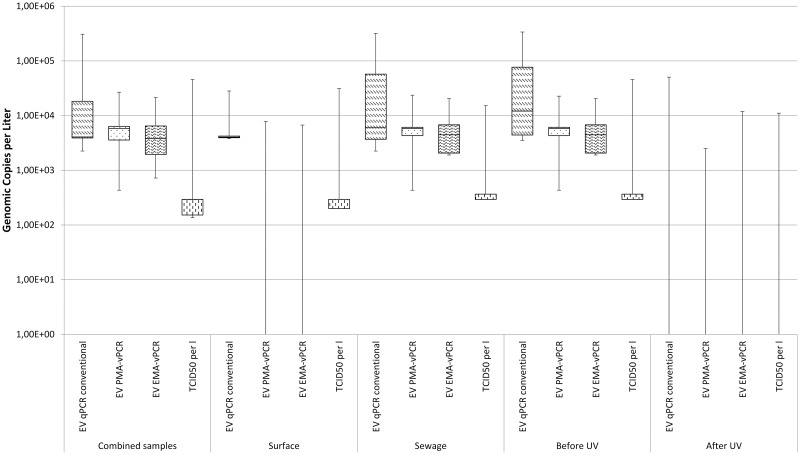
Boxplot comparison of the calculated concentration for enterovirus originating from surface waters and sewage waters before and after UV treatment as well as total samples.

The abundance of EV was remarkably lower than HAdV in all sampling categories. Less than 15% of the combined sewage and surface samples showed EV using RT qPCR (13/79) with a median concentration of 4.08E+03 GC/l, while pretreatment with EMA and PMA resulted in 6 positive samples for both substances with a median of 5.81E+03 GC/l (see Fig 3). Application of vPCR for surface water reduced the signals for RT-qPCR (6/53) and both dyes (EMA 2/53; PMA 2/53) while obtaining comparable median concentrations for both conventional and pretreated molecular detection.

Analysis of the combined sewage water samples before and after UV treatment with RT-qPCR showed an occurrence of EV in 7 out of 26 samples while EMA and PMA pretreatment lead to lower detection rates (4/26 for both dyes) and lower median GC/l.

Comparison between before and after UV disinfection indicates that EV—unlike HAdV—is more susceptible to UV disinfection. Conventional molecular quantification methods and viability RT-qPCR represented this with positive signals before and negative after the exposure.

### Molecular Quantification and Detection of Rotavirus A (RV)

**Fig 4:**
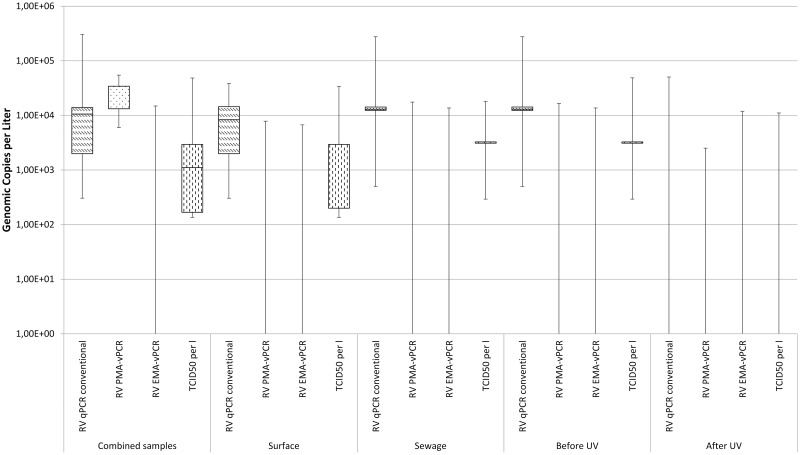
Boxplot comparison of the calculated concentration for rotavirus A originating from surface waters and sewage waters before and after UV treatment as well as total samples.

RT-qPCR showed a slightly higher occurrence and median genomic concentration per liter of RV compared to EV in all samples (see Fig 4). Almost 20% of the combined, surface and sewage water samples were positive using this method. Pretreatment with EMA lead to negative RV signals in the water samples regardless of the source while only small numbers of the combined (3/79), surface (2/53) and sewage water samples (1/26; only inflow) were positive for PMA treated samples.

UV disinfection seemed to be able to successfully inactivate RV in the treatment process which was visible by the outflow being negative with both conventional and PMA pretreated samples. Inflow showed positive results (5/13) via RT-qPCR and (1/13) using PMA pretreatment.

### Quantification and Detection of HAdV, EV and RV by TCID_50_ and ICC-qPCR

Inoculation of A549 cells with environmental samples previously positive in qPCR lead to CPE in around half of them (41/79) combined. Surface water samples (28/53) and sewage water (13/26) showed similar rates. The relatively high resistance of HAdV to UV light was previously described (46, 56) and could be also observed in this study since HAdV was positive before (7/13) and after UV treatment (4/13).

Both EV and RV showed considerably lower infectivity rates in sample categories. Both proved to be infectious in around 10% of the combined samples (EV 10/79; RV 8/79) previously positive by RT-qPCR. Analysis of the surface water samples showed comparable rates (EV 5/53; RV 6/53) and the efficacy of UV treatment for both enteric viruses was visible since no UV treated samples were positive while 38% (5/13) and 46% (6/13) showed CPE before exposure to UV for EV and RV; respectively.

### Cost and Time Comparison of Detection Methods

Application of the conventional molecular quantification methods for all viruses is the cheapest and fastest way to obtain results. The pretreatment of environmental samples with EMA and PMA before quantification adds 2 hours and 0.20 to 0.98 Euro in comparison to the qPCR and RT-qPCR. Prices in cost and time are much higher for both cell culture and ICC-qPCR as seen in Table 4.

**Table 4:** Material cost and time per reaction for each of the utilized methods (incl. sample preparation, excl. personal cost).

Method		Price per Reaction in Euro (incl. taxes; 25.04.2016)	Approximate time to get results per Reaction
**molecular method**	Quantitative PCR (qPCR)	4.76	4 hours
	Viability qPCR EMA	4.96	6 hours
	Viability qPCR PMA	5.72	6 hours
	Reverse Transcription (RT) qPCR	7.80 (12.09 for RV)	7 hours
	RT-qPCR EMA	8.00 (12.28 for RV)	9 hours
	RT-qPCR PMA	8.76 (13.05 for RV)	9 hours
**cell-culture based**	Integrated Cell Culture qPCR	20.26	3–5 days
	Integrated Cell Culture RT-qPCR	26.34 (30.63 for RV)	3–5 days
	Cell Culture TCID_50_	14.74	approx. 7 days

## Discussion

### Current Methods of Environmental Virology

Waterborne enteric viruses are a growing concern to public health in both the developed and developing world. Currently, a wide range of analytical methods is available for virus detection in environmental water samples, while most of the data collected and published is obtained by PCR and/or cell culture methods.

### Comparison of the Detection and Quantification of HAdV

The quantitative and qualitative detection of human adenovirus in both sewage and surface water is of great importance due to the fact that HAdV is discussed as a potential indicator of fecal water contamination [44]. HAdV is stable under most environmental conditions and survives UV-exposure in sewage treatment [34, 45, 46], it is shed in high concentrations and—unlike norovirus and rotavirus—shows almost no seasonality [47]. Detection of the virus by molecular and cell culture based methods is relatively easy due to the DNA genome and commercially available cell lines for viral propagation. Besides being a potential indicator, HAdV also poses a threat to public health since HAdV serotype 41 has the highest abundance in both surface and sewage water matrices and is known to be the second most common agent to cause infections of the gastro intestinal tract in children worldwide [48, 49].

In the presented work, HAdV has been detected in 85% of the surface water and 96% of the sewage water—in samples both before and after UV treatment. These detection rates concur with the literature in general [37, 38] and those obtained in our previous studies [36, 50, 51]. The detection rates also correspond to the detection of HAdV in cell culture confirmed by integrated cell culture since both methods showed no positive results if qPCR was negative. This adds to the assumption that the utilized primer set first published by Heim et al. [38] allows highly specific and sensitive HAdV detection but since the target region in the genome is highly conserved, it stays intact long after the virus is rendered non-infectious by environmental conditions.

Pretreatment of the samples positive in qPCR with either EMA and PMA showed that 52% of the combined, 40% of the surface- and 77% of the sewage water samples continued to show signals in the PMA-qPCR, 21% of the surface and 69% of the sewage water samples remained positive for EMA-qPCR. The cell culture based analysis of the same samples positive in qPCR lead to 52% positive for the combined, 53% for the surface water and 50% for the sewage water. Detection rates and virus concentrations for the surface and sewage water correspond to previously performed infectivity assays in the context of a long-term evaluation of the same river [36, 50].

### Comparison of the Detection and Quantification of Enteroviruses

Members of the family of enteroviruses are known to be relatively stable in the water phase at low to medium temperatures as shown by Prevost, Goulet [32]. Coxsackievirus was used to study the persistence of enteric RNA viruses in the environment and was like HAdV 41 able to remain infectious after more than 70 days at 4°C and 20°. Even though not as persistent towards UV exposure like HAdV, nucleic acid of the non-enveloped virus could be detected after inactivation with UV at doses up to 400 mJ/cm^2^ and 10 mg/min/l free chlorine.

Previous studies by Kahler, Cromeans [52] mimicked the effect of environmental inactivation and Kyriakopoulou, Dedepsidis [53] were able to identify enterovirus strains which were present in Larissa, Greece both before and after sewage treatment which indicates the stability of the virus.

Enterovirus has been found in the river under investigation before [3] using pan-entero primers and probes first developed by Hamza, Jurzik [40]. Both the detection rates and median concentrations in the presented work concur with previous findings.

Using qPCR, EV was detected in 17% of the combined, 11% of the surface and 27% of the sewage water samples. A higher susceptibility towards UV treatment in comparison to HAdV could be observed since 46% of the inflow and only 8% of the outflow samples were positive for EV using qPCR. PMA-qPCR resulted in merely 4% positives in surface, 15% in sewage water samples and 8% in the combined. The same results were obtained for EMA-qPCR.

Cell culture based analysis of the samples indicates a slight risk of overestimating qPCR results and at the same time the inadequacy of relying solely on viability qPCR with 13% of the combined, 9% of the surface and 15% of the sewage water samples showing CPE.

Application of EMA-/PMA-qPCR for sewage water succeeds in showing inactivation of enterovirus by UV exposure, though- sewage outflow samples stay positive in one thirds of the cases (31%) which is in accordance with infectivity detected by cell culture (39%). No signals for qPCR have been shown for the outflow using all methods evaluated.

### Comparison of the Detection and Quantification of Rotavirus A

Gastroenteritis and diarrhea caused by rotavirus infections pose a significant threat to especially the health of newborns and immunosuppressed in low- and middle income countries [54, 55]. The rotavirus vaccine for infants recommended by WHO in 2013 has the potential to counter that danger but until a herd effect is reached [56], RV remains one of the most relevant waterborne enteric viruses.

The German federal ministry of research and education (BMBF) recently conducted Safe Ruhr, a long term project to investigate the suitability of an urban river in the Rhine-Ruhr area in North Rhine Westphalia, Germany for recreational activity. Results from this project indicated that even though RV is detected in less than 15% of the samples over a duration of three years using RT-qPCR it causes the highest amount of disability adjusted life years (DALY) for recreational activity in said lake [50].

Detection rates and median concentrations of RV in the presented work slightly exceed those of EV for combined and surface water with 19% positives in the overall and 19% positives in the surface water but remain slightly lower for sewage water samples (19% compared to 27% for EV).

Pretreatment with the intercalating dyes showed that RV remains detectable only after application of PMA in 4% of combined, surface and sewage and 8% of the sewage inflow.

Cell culture based methods indicate an imprecision and the risk of false negative results in the analysis of viral infectivity as previously observed for enterovirus: 10% of the combined, 11% of the surface and 8% of the sewage water samples showed cytopathic effects. Sewage water inflow also shows RV in six times more samples (46% for cell culture compared to 8% for PMA-qPCR and no positives for EMA-qPCR) which concurs with the postulated relative vulnerability of RV towards UV exposure [57].

### Disadvantages of the EMA/PMA-qPCR for Enteric Viruses

Unlike HAdV the detection of the analyzed RNA viruses EV and RV require the reverse transcription of the extracted sample into complementary DNA. Organic substances are known to interfere with this step in the processing of environmental samples and thereby can reduce the precision and robustness of the detection in direct comparison to DNA viruses like HAdV [58] which could explain the shift in the detection rates.

Several publications have been released in which different adaptions for the application of EMA/PMA for RNA viruses like norovirus have been proposed [30, 59]. Complex matrices like sewage and surface water require special treatment though before the pretreatment is possible. Due to the different capsid characteristics, it may also be advised to develop standard virus exposure times and EMA/PMA concentrations for the most relevant enteric viruses.

## Conclusion

Pretreatment using both PMA and EMA appears to be capable to reduce false positive qPCR signals obtained by conventional molecular methods. Application of both reagents therefore indicates a potential to allow a more realistic evaluation of the viral load of complex environmental water matrices for the highly abundant HAdV and to a certain extent for the RNA viruses of the EV group and RV. To represent the mode of virus inactivation and therefore the distinction of infectivity analyzed, it is proposed to rename the viability qPCR to capsid integrity qPCR (ci-qPCR).

Considering the comparably lower price per reaction for both dyes in time and cost (with EMA in particular) and the fact that pretreatment with the reagents requires significantly less experience and facilities than cell culture assay, their application in low resource settings can help overcome the monetary barrier which inhibits the much needed evaluation of environmental water bodies in many regions worldwide.

## Supporting Information

S1 File **Table A. Unprocessed Data for human Adenovirus.** Median, 25-/75-percentile and minimum/maximum for all sampling sites and types **Table B. Unprocessed Data for enterovirus.** Median, 25-/75-percentile and minimum/maximum for all sampling sites and types **Table C. Unprocessed Data for rotavirus.** Median, 25-/75-percentile and minimum/maximum for all sampling sites and types.
(XLSX)
